# Teprotumumab for the treatment of chronic thyroid eye disease

**DOI:** 10.1038/s41433-021-01593-z

**Published:** 2021-07-09

**Authors:** Shoaib Ugradar, Julia Kang, Andrea L. Kossler, Erin Zimmerman, Jenna Braun, Andrew R. Harrison, Swaraj Bose, Kimberly Cockerham, Raymond S. Douglas

**Affiliations:** 1 The Jules Stein Eye Institute, University of California, Los Angeles, CA USA; 2 Central Valley Eye Medical Group, Stockton, CA USA; 3 Byers Eye Institute, Stanford University School of Medicine, Palo Alto, CA USA; 4 Cedars-Sinai Medical Centre, Los Angeles, CA USA; 5 Departments of Ophthalmology and Visual Neuroscience and Otolaryngology, University of Minnesota, Minneapolis, MN USA

**Keywords:** Autoimmune diseases, Outcomes research

## Abstract

**Background:**

Teprotumumab, a novel IGF-1R antibody was recently shown to significantly reduce the signs of active Thyroid eye disease (TED). The current study reviews its efficacy in chronic TED.

**Methods:**

In this retrospective review, consecutive patients with chronic stable TED (>2 years), who had received ≥3 infusions of teprotumumab were included. All patients had measurements of proptosis, and calculation of the CAS and diplopia scores before and after therapy. Five-point strabismus scores were also calculated. Patients who had imaging within 4 months prior to therapy and 6 weeks post therapy underwent orbital 3D volumetric analysis.

**Results:**

Thirty-one patients met the inclusion criteria. The mean (SD) duration of TED was 81 months (56) and the mean (SD) number of infusions received by each patient was 7 (2). Mean (SD) reduction in proptosis for each study orbit was 3.5 mm (0.4) and 3 mm (0.3) for the fellow orbit. The CAS response was 90% for the study orbit and 87% for the fellow orbit. Of the 15 patients who had diplopia at baseline, 67% had a clinically significant response, while 47% had complete resolution following treatment. Following teprotumumab, mean (SD) reduction of muscle tissue was 2011 mm^3^ (1847) in the study orbit and 1620 mm^3^ (1759) in the fellow orbit. The mean (SD) reduction of fat volume was 2101 mm^3^ (1681) in the study orbit and 1370 mm^3^ (1181) in the fellow orbit.

**Conclusion:**

Teprotumumab significantly reduces proptosis, inflammation, diplopia, strabismus and orbital soft tissue volume in patients with chronic TED.

Thyroid eye disease (TED) is a complex, debilitating autoimmune condition most commonly associated with Graves’ disease that can cause significant disfigurement, vision loss, and psychosocial sequelae [1]. Approximately 40% of patients with Graves’ disease (GD) will develop ocular involvement with signs and symptoms that range from mild to severe [2]. At presentation, common symptoms include pain, diplopia, tearing, redness and decreased vision [1]. Exam findings may include dry eye, eyelid retraction, proptosis, lagophthalmos, extraocular dysmotility, inflammation and oedema of the periorbita and conjunctiva, and signs of optic nerve compromise [1]. Classic radiographic findings include orbital fat expansion [3], enlargement of the extraocular muscles and expansion of the bony orbital cavity [4].

The current consensus suggests that the natural history of the disease is biphasic. It begins with an active phase with inflammatory signs and/or soft tissue expansion followed without resolution, by a chronic phase characterised by fibrosis with a lack of further soft tissue expansion and a reduction of inflammatory signs [5]. Conventional management for the active phase has been thus far limited almost exclusively to immune suppression with agents such as corticosteroids [6, 7]. Present day treatments have failed to inhibit orbital fibroblast (OF) activation, which is responsible for the majority of soft tissue expansion through the production of extracellular matrix (ECM) proteins [8]. Since immune activation forms only one facet of disease progression, these treatments have been shown to reduce inflammatory signs but not significantly alter proptosis [9]. Similarly, some second-line immunomodulatory agents such as Rituximab have been found to be potentially useful in the inflammatory phase, but with limited effect on long-term disease outcomes such as proptosis and diplopia [10]. Given the underlying pathophysiologic event in TED is OF activation resulting in soft tissue expansion, differentiation to fat cells and myofibroblasts in conjunction with hyaluronic acid (HA) production, debulking treatment, such as decompression procedures have been the mainstay of therapy for proptosis in the chronic non inflammatory phase [11].

A key pathological feature of TED, regardless of disease phase, is the overexpression of the insulin-like growth factor 1 receptor (IGF-1R) and its interaction with the thyrotropin receptor (TSH-R) [12]. Both receptors form a functional complex on the cell membrane of OFs, B cells and T cells [13]. In TED, autoantibodies to IGF-1R bind to the complex, leading to increased production of the proinflammatory cytokines [14], IL-2 [15], TNF-α [16], and IL-8 [17] by T cells and monocytes and hyaluronan by OFs [18]. In the chronic phase the overexpression of the receptor on these tissues persists [19]. This persistent overexpression is likely associated with the sustained alteration in metabolic profile of OFs, sustaining the increased tissue expansion. Fundamentally the overexpression of the IGF-1R in both the active and chronic disease phases appears to be a key pathophysiologic mechanism for disease [8].

Teprotumumab, a fully human monoclonal immunoglobulin specifically binds and blocks signal transduction of the IGF-1R and IGF-1R/TSHR complex on orbital fibroblasts. Teprotumumab, was recently approved by the FDA for the treatment of TED in the US [20]. In previous phase 2 and 3 randomised, double-masked clinical trials (NCT01868997 and NCT03298867) [21, 22] teprotumumab was more effective than placebo in reducing proptosis, diplopia and inflammation in patients with active TED [21]. In both trials, only patients with ocular symptoms that presented within 9 months of baseline assessment with a clinical activity score (CAS) of 4 or greater were enroled.

However, there is a paucity of literature regarding the efficacy of teprotumumab in the treatment of chronic, non-inflammatory TED. At the time of writing, one case report demonstrated marked improvement in proptosis in a patient with TED for 3 years [23]. Given the sustained increased expression of IGF-1R on orbital tissues in chronic TED, teprotumumab may likely improve proptosis even after many years of clinical stability.

In this study, we review the response to teprotumumab therapy in consecutive patients who have had chronic (>2 years) TED. The primary outcome measures were proptosis reduction and orbital soft tissue volume measurement, while secondary outcome measures included the CAS and measures of strabismus and diplopia.

## Methods

This study adhered to the tenets of the Declaration of Helsinki, was performed in accordance with the Health Insurance Portability and Accountability Act (HIPAA) and was approved by the sites’ institutional review boards. All patients provided written consent for the studies.

### Patients

This was a multicentre study, with patients being recruited from four separate TED centres across the US. Patients that presented to our institutions for the treatment of symptomatic TED were considered for study eligibility. Consecutive patients who had been diagnosed with TED for more than 2 years, had a clinical activity score (CAS) ≤ 3 without any changes in proptosis or diplopia over the preceding year as determined by the patient and physician and received ≥3 infusions of teprotumumab, were included in the study. Patients who were on any other medical therapy for TED or had received Rituximab or Tocilizumab in the past were excluded. Patients received infusions of teprotumumab (10 mg/kg for the first infusion and 20 mg/kg for subsequent infusions) every 3 weeks with the intention to complete 8 infusions over 24 weeks.

### Measurement of clinical outcomes

The primary outcome measures were a proptosis response (defined as a reduction in proptosis of ≥2 mm from baseline in the study eye without a corresponding increase of ≥2 mm in the fellow eye) following the last infusion and changes to orbital soft tissue following treatment. A reduction of ≥2 mm in proptosis was considered clinically significant. Proptosis was assessed using the same Hertel exophthalmometer by the same person at each centre, per visit.

Key secondary outcomes included the mean change in proptosis in millimetres in the study eye between baseline and the final infusion, a diplopia response (defined as a reduction in diplopia of ≥1 grade from baseline) following the last infusion and the response rate on the CAS of 0 or 1 (indicating no or minimal inflammation, respectively) at the latest visit.

Changes in diplopia grade were assessed using the Gorman subjective diplopia score [6] (range 0–3). A score of 0 indicates no diplopia; 1, intermittent diplopia; 2, inconstant diplopia and 3, constant diplopia. An improvement ≥1 grade is considered clinically significant. Further, consecutive patients from two centres also had 4 gaze measurements with photographic documentation to subjectively document dysmotility. All pre and post gaze assessments were carried out by the same person and using a five-point scale (for any gaze direction, 0 = no restriction, −1 = 25% restriction, −2 = 50% restriction, −3 = 75% restriction and −4 = no movement).

Inflammation was assessed using the 7-point CAS [24], which scores the presence of each of the following signs: retrobulbar eye pain, pain on eye movement, eyelid erythema, eyelid swelling, conjunctival redness, chemosis, inflammation of the caruncle or plica. A CAS ≤ 1 is indicative of disease inactivation [24].

### Image analysis

Previous work has shown that fat and muscle volume reduce in patients with acute TED who are treated with teprotumumab [25]. Volumetric analysis was used in the present study to determine if similar changes occurred in patients with chronic TED who were treated with teprotumumab. Patients were included for this analysis if they had imaging prior to therapy and within 6 weeks post therapy. Further, only patients with sub 1 mm imaging slices were included.

Three-dimensional volumetric analyses of the orbital fat and muscle volume were performed using the previously validated 3D image analysis software, MIMICS (Materialise, Leuven, Belgium) [26].

Soft tissue measurements were calculated using the technique previously described by Ugradar et al. [3]. Quasicoronal images were rotated as necessary to align the midline of the brain to vertical and thereby eradicating any potential for errors from head tilting during the scan. Using the software, Hounsfield units (HUs) of fat and muscle tissue were defined manually. Using the axial image, a horizontal line was drawn across the junction of the globe and the optic nerve. All muscle tissue between this line and the orbital outlet of the optic canal was included. Fat was measured between the septum and the orbital outlet of the optic canal. A mask was created by manual segmentation, slice by slice. A 3D model created by voxel addition was expressed in millimetres cubed. Segmentation and measurements were performed independently by two graders to review repeatability.

### Statistical analyses

Statistical analysis was performed using SPSS version 22.0 (SPSS, Inc, Chicago, Illinois, USA). Differences in proptosis measurements before and after commencing therapy were assessed using a dependent *t* test, while the differences between CAS and diplopia scores at different time points were analysed using the Wilcoxon Signed Rank test. Due to the occurrence of asymmetric TED, each orbit was treated as a separate entity. Statistical significance was defined as *p* < 0.05. In each patient, a single orbit was designated the study orbit, which was based on the more severely affected orbit in terms of proptosis, in accordance with previous clinical trial protocols [21, 22]. In cases where exophthalmometry was equal in both orbits, one was chosen at random. For interobserver variability, intraclass correlation coefficients and their 95% confidence intervals were calculated. The magnitude of the measurement error between the observers was calculated using the Bland Altman method.

## Results

### Patients

A total of 31 consecutive patients (8 males, 23 females) met the inclusion criteria. The mean (SD) age was 57 (16). The mean (SD) duration of TED prior to treatment was 81 months (56). The mean (SD) number of infusions received by each patient was 7 (2). All patients were euthyroid and none of the patients were current smokers. Further, 55% (17/31) of patients had completed 8 infusions of teprotumumab therapy at the time of analysis. No patients discontinued therapy due to adverse events.

## Clinical outcomes

### Proptosis

**Fig. 1:**
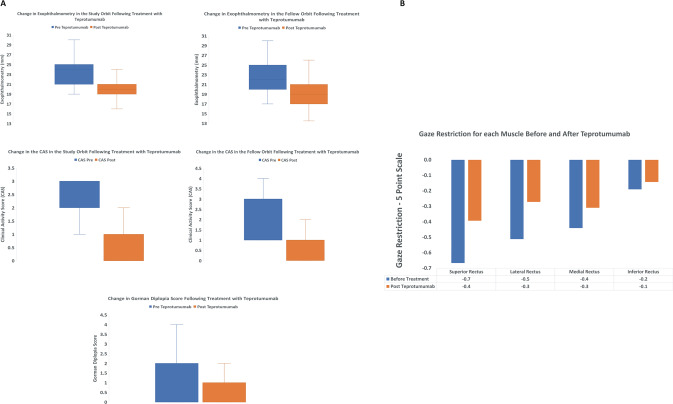
Clinical measurements before and after Teprotumumab. **a** Changes in exophthalmometry, the CAS and Gorman diplopia scores before and after therapy. **b** Changes to gaze restriction for each of the rectus muscles before and after Teprotumumab therapy.

Prior to therapy, the mean (SD) exophthalmometry in the study orbit was 24 mm (3.8) and 21 mm (4) following therapy (*p* < 0.01). In the fellow orbit, at baseline, mean (SD) exophthalmometry was 23 (4) and 20 mm (4) following therapy (*p* < 0.01). Mean (SD) reduction in proptosis for each study orbit was 3.5 mm (0.4) and 3 mm (0.3) for the fellow orbit. Ninety percent of study orbits had a clinically significant (≥2 mm) reduction in proptosis, while 84% of fellow orbits had a significant reduction in proptosis. Prior to therapy, five patients had asymmetric disease (proptosis difference ≥3 mm between orbits), while after treatment, four patients had asymmetric disease (Fig. 1a and Table 1).

**Table 1:** Clinical characteristics of patients pre and post therapy.

Case	VA OD pre	VA OS pre	VA OD post	VA OS post	CAS OD pre	CAS OS pre	CAS OD post	CAS OS post	Hertel OD pre	Hertel OS pre	Hertel OD post	Hertel OS post	Gorman score Pre	Gorman score post
**1**	20/30	20/100	20/30	20/100	2	3	1	1	18	19	16	14	4	2
**2**	20/50	20/30	20/30	20/30	3	4	1	1	23	22	20	19	0	0
**3**	20/25	20/20	20/25	20/20	3	3	2	2	21	21	20	20	4	4
**4**	20/20	20/20	20/20	20/20	1	3	0	0	20	25	17	20	1	1
**5**	20/20	20/20	20/20	20/20	2	2	0	0	19	21	17	18	1	1
**6**	20/25	20/25	20/25	20/25	3	3	0	0	24	24	20	20	0	0
**7**	20/20	20/20	20/20	20/20	2	2	0	0	21	24	19	21	3	0
**8**	20/20	20/20	20/20	20/20	3	3	1	1	20	21	18	19	0	0
**9**	20/20	20/20	20/20	20/20	3	3	1	1	23	24	18	19	0	0
**10**	20/20	20/20	20/20	20/20	1	1	1	1	25	27	22	22	0	0
**11**	20/40	20/50	20.40	20/50	1	2	0	0	20	20	19	19	0	0
**12**	20/20	20/20	20/20	20/20	1	1	0	0	20	21	18	19	0	0
**13**	20/20	20/20	20/20	20/20	1	1	0	0	20	21	18	19	0	0
**14**	20/25	20/40	20/30	20/25	1	1	1	1	28	27	26	25	4	4
**15**	20/30	20/30	20/30	20/30	3	3	1	1	25	25	23	21	0	0
**16**	20/30	20/25	20/25	20/25	3	3	0	0	18	19	17	17	2	1
**17**	20/20	20/30	20/20	20/20	2	2	0	0	29	28	25	26	1	0
**18**	20/80	20/25	20/80	20/25	2	2	1	1	28	25	20	17	2	1
**19**	20/25	20/20	20/20	20/20	3	3	0	0	25	25	24	24	0	0
**20**	20/25	20/20	20/25	20/20	1	1	0	0	22	20	19	17	2	0
**21**	20/25	20/25	20/25	20/40	1	1	0	0	30	30	22	25	0	0
**22**	CF	HM	20/400	CF	3	0	0	0	24	30	15	25	0	0
**23**	HM	HM	CF	HM	3	3	1	1	35	35	33	31	0	0
**24**	20/20	20/20	20/20	20/20	3	3	0	0	21	22	19	19	2	0
**25**	20/25	20/60	20/20	20/40	3	3	0	0	17	23	13.5	16	3	3
**26**	20/60	20/50	20/50	20/40	2	2	0	0	24	24	20	21	2	0
**27**	20/40	20/70	20/25	20/25	3	3	0	0	24.5	24	20.5	20.5	0	0
**28**	20/40	20/30	20/25	20/30	3	3	2	2	23	24	20	21	0	0
**29**	20/40	20/20	20/20	20/40	3	3	2	2	20	20.5	17	18	3	0
**30**	20/30	20/30	20/30	20/30	3	3	0	0	32	32.5	26	28	2	0
**31**	20/20	20/25	20/25	20/25	3	1	3	1	18	19	18	15	0	0

*VA* visual acuity, *CAS* clinical activity score.

### Clinical activity score

In the study orbit, mean (SD) CAS was 2.3 (0.9) before therapy and 0.5 (0.7) following therapy (*p* < 0.01). The mean (SD) reduction in CAS was 1.8 (1). In the fellow orbit, mean (SD) CAS was 2.3 (0.9) before therapy and 0.5 (0.8) following therapy (*p* < 0.01). The mean (SD) reduction in CAS was 1.8 (1). For the study orbits, the CAS response was 90% for the study orbit (28/31) and 87% (27/31) for the fellow orbit (Fig. 1a and Table 1).

### Diplopia

At baseline, the mean (SD) Gorman score was 1 (1.4) and improved to 0.5 (1) following therapy (*p* < 0.05) (Fig. 1a and Table 1). From the 15 patients who had diplopia at baseline, 10 (67%) had a clinically significant response, while 7 (47%) patients had complete resolution following treatment (Fig. 1a and Table 1). Using a 5-point strabismus scale, the greatest reduction in strabismus was seen in upgaze. Prior to therapy, the mean (SD) strabismus score for upgaze (superior rectus) was −0.7 (1) and −0.4 (1) following therapy (*p* < 0.01). Before therapy, the mean (SD) strabismus score for downgaze (inferior rectus) was −0.19 (0.7) and −0.14 (0.7) following therapy (*p* = 0.16). The mean (SD) score for gaze associated with the lateral rectus was −0.5 (1) prior to therapy and −0.3 (0.7) following therapy (*p* < 0.05). Finally, the score for gaze associated with the medial rectus was −0.5 (0.8) prior to therapy and −0.3 (0.7) following therapy (*p* = 0.08) (Fig. 1b).

### Orbital imaging analysis

Fifteen patients (30 orbits) included in the study had pre and post treatment imaging and were therefore included for volumetric analysis. All patients who had CT scans within 4 months prior to therapy and 6 weeks post therapy were included.

### Extraocular muscle volume

The mean (SD) muscle volume within the study orbit prior to therapy was 6598 mm^3^ (3716). Post-therapy, mean (SD) muscle volume significantly reduced to 4587 mm^3^ (2228) (*p* < 0.01). For the fellow eye, prior to therapy, mean (SD) muscle volume was 6244 mm^3^ (3578), while the mean (SD) volume post therapy was 4625 mm^3^ (2227) (*p* < 0.01). Mean (SD) reduction of muscle tissue was 2011 mm^3^ (1847) in the study orbit and 1620 mm^3^ (1759) in the fellow orbit.

### Orbital fat volume

**Fig. 2:**
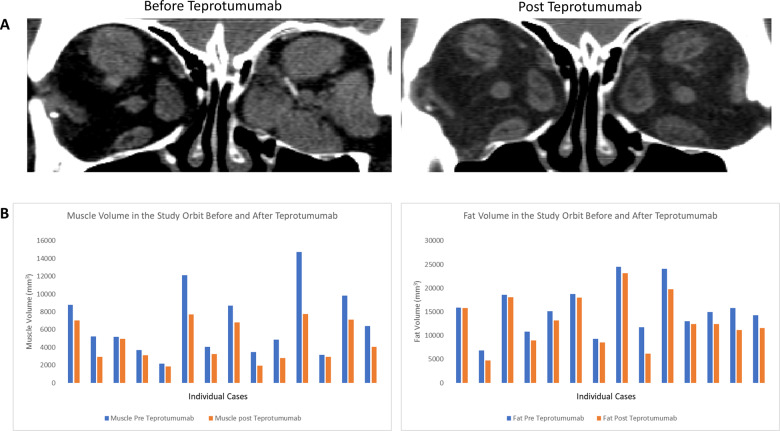
Changes to orbital soft tissue before and after Teprotumumab therapy. **a** CT scan showing the extraocular muscles before and after completion of teprotumumab therapy in the same patient. **b** Extraocular muscle and orbital fat volume in each patient before and after Teprotumumab therapy.

The mean (SD) fat volume within a single study orbit prior to therapy was 15,243 mm^3^ (5043). Post-therapy, mean (SD) fat volume significantly reduced to 13,142 mm^3^ (5286) (*p* < 0.01) (Fig. 2). For the fellow eye, the mean (SD) fat volume was 14,372 mm^3^ (4086) prior to therapy and 13,002 mm^3^ (4276) post therapy (*p* < 0.01). Mean (SD) reduction of fat volume was 2101 mm^3^ (1681) in the study orbit and 1370 mm^3^ (1181) in the fellow orbit.

### Reliability of measurements

Interobserver variability revealed a strong correlation between two observers for measurement of muscle volume (0.99) and fat volume (0.98).

### Descriptive case—case 30

**Fig. 3:**
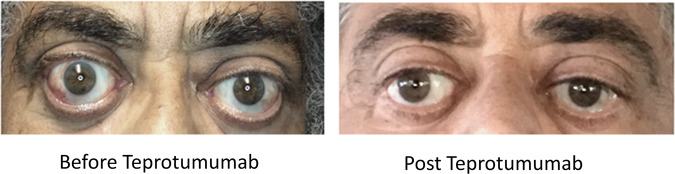
Clinical case. Photograph at baseline and following treatment with teprotumumab.

A 68-year-old black male with a 9 year history of Graves’ orbitopathy presented with progressively worsening proptosis and lagophthalmos. He had previously been treated with steroids, bilateral orbital radiation, bilateral orbital decompression and strabismus surgery. On presentation, his visual acuity was 20/30 OU. He had no signs of optic neuropathy, his colour vision was formally tested and found to be normal. Hertel exophthalmometry measurements were 32 mm OD and 32.5 mm OS. His CAS was 3 OU. His diplopia score was 2 with gaze restriction involving the right eye (−1 looking right and −2 looking left). Following treatment with Teprotumumab (8 infusions), his proptosis reduced to 26 mm OD and 28 mm OS. His diplopia score reduced to 0 and gaze restriction improved. His CAS reduced to 0 in both eyes (Fig. 3).

## Discussion

**Fig. 4:**
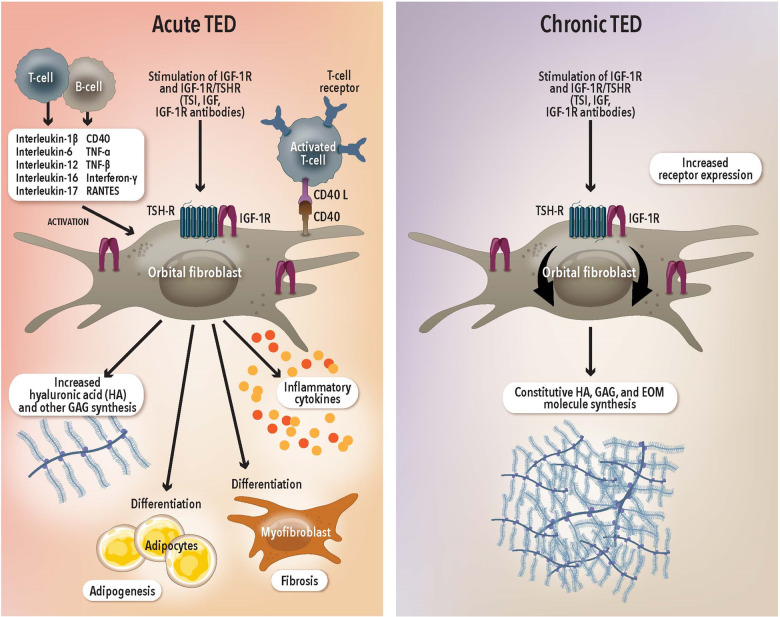
The role of orbital fibroblasts (OFs) in acute and chronic TED. The IGF-1R is overexpressed on OFs in the acute and chronic phase. In the acute phase, activation of the IGF-1R/TSHR pathway leads to proliferation of inflammatory cytokines, production of hyaluronan and other extracellular matrix proteins and differentiation into adipocytes or myofibroblasts, resulting in tissue expansion. In the chronic phase, the overexpression of IGF-1R on OFs persists and maintains tissue expansion. RANTES regulated upon activation, normal T cell expressed, and secreted, also known as CCL5, HA hyaluronic acid, GAGs Glycosaminoglycans.

In two placebo controlled randomised clinical trials, teprotumumab has been shown to be a promising and well tolerated first line treatment for TED [21, 22]. However, the previous studies have been limited to patients with ocular involvement for <9 months to capture the “active” inflammatory phase of the disease. In recent work, we showed that the IGF-1R is overexpressed on OFs in patients with active and chronic TED [19]. A key effector in the pathogenesis of TED is the OF. Activation of the IGF-1R initiates intracellular signalling along the Akt signalling pathway, a stimulator of cell growth and proliferation, and a potent inhibitor of programmed cell death [27]. In GD, OFs that are stimulated by IGF-1 have an increased propensity toward HA production and proliferation of extracellular matrix proteins, contributing significantly to the anabolic effects seen in TED (Fig. 4) [14]. Hydration of hyaluronan within orbital tissue leads to swelling and soft tissue expansion within a rigid bony cavity, causing proptosis.

Based on this data, our hypothesis is teprotumumab may be effective in patients with chronic TED. Aside from a case description [23] there have been no clinical series providing clinical evidence to support a potential role for teprotumumab in the treatment of chronic TED.

The present study describes the experience of 31 consecutive patients with chronic TED, treated with teprotumumab. These patients presented, on average, 7 years after being diagnosed with TED and demonstrated a marked improvement in proptosis, CAS and diplopia. Ninety percent of study orbits had a clinically significant improvement (≥2 mm) in proptosis, while 84% of fellow orbits also had a clinically significant improvement. Further, there was a significant reduction in fat and muscle volume following treatment, which may explain some of the reduction in proptosis. Sixty seven percent of patients experienced a clinically significant improvement in diplopia while 47% had complete resolution following therapy.

Given the IGF-1R controls the constitutive metabolic turnover of macromolecules in the extracellular matrix by OFs [28], the recent finding of overexpression of the IGF-1R in longstanding disease becomes more meaningful in the context of teprotumumab [19]. Despite the dormant appearance of chronic TED, OFs, continually turnover HA and other ECM macromolecules once a week to maintain tissue integrity [28]. Therefore, interrupting the IGF-1R pathway may reduce the downstream signalling that leads to tissue expansion in chronic TED (Fig. 4).

In blocking the IGF-1R, teprotumumab may reduce the activity of this pathway, causing a reduction in soft tissue volume within the orbit. This notion gains support from a previous study in which patients with active TED were treated with teprotumumab, leading to a marked reduction in extraocular muscle volume [25].

The results of the present study raise key questions regarding the potential for widening access to teprotumumab for patients with chronic TED, most pertinently, in countries with a state sponsored healthcare system. A previous report on the economic and public health impact of TED revealed that on average, 22% of patients were registered as temporarily disabled, while 6% were registered as permanently disabled [29]. This study was conducted in Germany, an economy with a predominantly state funded healthcare system, much like the UK. The total indirect economic burden of TED was calculated at $3,475,325,640. When adjusted for inflation since 2013, this figure rises to $3,951,518,218 (£2,502,060,694) in 2021 [29].

The burden on the quality of life of patients with TED is also significant. Ponto and colleagues also reported that from a pool of 250 outpatients in a TED clinic, 45% complained of restrictions in their daily activities, 38% reported impaired self-perception, 21% underwent psychotherapy and 36% were on sick leave because of their TED [30].

Given the significant impact of TED on the economy and quality of life, an extensive cost benefit analysis for the use of teprotumumab in patients with chronic disease is encouraged.

The limitations of the present study pertain to the inclusion of patients with a heterogenous history of TED. Some patients had surgical intervention, while others had only medical therapy prior to inclusion. Although the study focused on the worse affected orbit (study orbit), this may have implications for the magnitude of the effect of teprotumumab in our group of patients. On the other hand, a key strength of the study was its longitudinal design, allowing for a robust comparison within patients. Long term follow-up to review longevity of treatment impact was not available for this study and is the subject of ongoing work at our institutions.

There is a growing body of evidence, which suggests that chronic TED may not be as inactive as previously thought. The present study adds to that and prompts a discussion regarding potentially widening the group of patients who are deemed to potentially benefit from teprotumumab.

### Summary

#### What was known before

- Teprotumumab clinically improves the signs and symptoms of Thyroid eye disease in patients with active disease of recent onset (<2 years).

#### What this study adds

- Teprotumumab also has a clinically significant impact on patients with stable chronic (>2 years), significantly reducing proptosis, diplopia, inflammation and strabismus.

## Supplementary information

Table 2

Table 3

Table 4

## References

[1] Bahn RS (2010). Graves’ ophthalmopathy. N. Engl J Med.

[2] Abraham-Nordling M, Byström K, Törring O, Lantz M, Berg G, Calissendorff J (2011). Incidence of hyperthyroidism in Sweden. Eur. J. Endocrinol..

[3] Ugradar S, Rootman DB. Orbital fat expansion in thyroid eye disease is related to age. Eur. J. Ophthalmol. 2020;30. 10.1177/1120672119852322 31144509

[4] Ugradar S, Goldberg RA, Rootman DB (2019). Bony orbital volume expansion in thyroid eye disease. Ophthal. Plast. Reconstr. Surg..

[5] Rundle FF, Wilson CW (1945). Development and course of exophthalmos and ophthalmoplegia in Graves’ disease with special reference to the effect of thyroidectomy. Clin. Sci..

[6] Bartalena L, Baldeschi L, Dickinson A, Eckstein A, Kendall-Taylor P, Marcocci C (2008). Consensus statement of the European Group on Graves’ orbitopathy (EUGOGO) on management of GO. Eur. J. Endocrinol..

[7] Bradley EA, Gower EW, Bradley DJ, Meyer DR, Cahill KV, Custer PL (2008). Orbital radiation for graves ophthalmopathy. A Report by the American Academy of Ophthalmology. Ophthalmology.

[8] van Steensel L, Dik WA (2010). The orbital fibroblast: a key player and target for therapy in Graves’ ophthalmopathy. Orbit.

[9] van Geest RJ, Sasim IV, Koppeschaar HPF, Kalmann R, Stravers SN, Bijlsma WR (2008). Methylprednisolone pulse therapy for patients with moderately severe Graves’ orbitopathy: A prospective, randomized, placebo-controlled study. Eur. J. Endocrinol..

[10] Pasquier-Fediaevsky LD, Andrei S, Berche M, Leenhardt L, Héron E, Rivière S (2014). Low dose of rituximab for corticosteroid-resistant graves’ orbitopathy. Eur. Thyroid J..

[11] Jefferis JM, Jones RK, Currie ZI, Tan JH, Salvi SM. Orbital decompression for thyroid eye disease: methods, outcomes, and complications. Eye. 2017. http://www.nature.com/doifinder/10.1038/eye.2017.260. 10.1038/eye.2017.260 PMC5848288 29243735

[12] Tsui S, Naik V, Hoa N, Hwang CJ, Afifiyan NF, Sinha Hikim A (2008). Evidence for an association between thyroid-stimulating hormone and insulin-like growth factor 1 receptors: a tale of two antigens implicated in Graves’ disease. J. Immunol..

[13] Krieger CC, Neumann S, Place RF, Marcus-Samuels B, Gershengorn MC (2015). Bidirectional TSH and IGF-1 receptor cross talk mediates stimulation of hyaluronan secretion by Graves’ disease immunoglobins. J. Clin. Endocrinol. Metab..

[14] Smith TJ, Hoa N (2004). Immunoglobulins from patients with graves’ disease induce hyaluronan synthesis in their orbital fibroblasts through the self-antigen, insulin-like growth factor-I receptor. J. Clin. Endocrinol. Metab..

[15] Kooijman R, Rijkers GT, Zegers BJM (1996). IGF-I potentiates interleukin-2 production in human peripheral T cells. J. Endocrinol..

[16] Renier G, Clément I, Desfaits AC, Lambert A (1996). Direct stimulatory effect of insulin-like growth factor-I on monocyte and macrophage tumor necrosis factor-α production. Endocrinology.

[17] Kooijman R, Coppens A, Hooghe-Peters E (2003). IGF-I stimulates IL-8 production in the promyelocytic cell line HL-60 through activation of extracellular signal-regulated protein kinase. Cell. Signal.

[18] Gillespie EF, Smith TJ, Douglas RS (2012). Thyroid eye disease: towards an evidence base for treatment in the 21st century. Curr. Neurol. Neurosci. Rep..

[19] Ugradar S, Shi L, Wang Y, Mester T, Yang H, Douglas RS. Teprotumumab for non-inflammatory thyroid eye disease (TED): evidence for increased IGF-1R expression. Eye. 2020. 10.1038/s41433-020-01297-w. Online ahead of print. 10.1038/s41433-020-01297-w PMC8376861 33221815

[20] Markham A (2020). Teprotumumab: first approval. Drugs.

[21] Smith TJ, Kahaly GJ, Ezra DG, Fleming JC, Dailey RA, Tang RA (2017). Teprotumumab for thyroid-associated ophthalmopathy. N. Engl. J. Med..

[22] Douglas RS, Kahaly GJ, Patel A, Sile S, Thompson E, Perdok R (2020). Teprotumumab for the treatment of active thyroid eye disease. N. Engl. J. Med..

[23] Ozzello DJ, Kikkawa DO, Korn BS (2020). Early experience with teprotumumab for chronic thyroid eye disease. Am. J. Ophthalmol. Case Rep..

[24] Mourits MP, Koornneef L, Wiersinga WM, Prummel MF, Berghout A, van der Gaag R (1989). Clinical criteria for the assessment of disease activity in Graves’ ophthalmopathy: a novel approach. Br. J. Ophthalmol..

[25] Jain AP, Gellada N, Ugradar S, Kumar A, Kahaly G, Douglas R. Teprotumumab reduces extraocular muscle and orbital fat volume in thyroid eye disease. Br. J. Ophthalmol. 2020. 10.1136/bjophthalmol-2020-317806 33172865

[26] Regensburg NI, Kok PHB, Zonneveld FW, Baldeschi L, Saeed P, Wiersinga WM (2008). A new and validated CT-based method for the calculation of orbital Soft tissue volumes. Investig. Ophthalmol. Vis. Sci..

[27] Hoa N, Tsui S, Afifiyan NF, Hikim A, Li B, Douglas RS (2012). Nuclear targeting of IGF-1 receptor in orbital fibroblasts from Graves’ disease. PLoS One.

[28] Kaback LA, Smith TJ (1999). Expression of hyaluronan synthase messenger ribonucleic acids and their induction by interleukin-1β in human orbital fibroblasts: Potential insight into the molecular pathogenesis of thyroid-associated ophthalmopathy. J. Clin. Endocrinol. Metab..

[29] Ponto KA, Merkesdal S, Hommel G, Pitz S, Pfeiffer N, Kahaly GJ (2013). Public health relevance of graves’ orbitopathy. J. Clin. Endocrinol. Metab..

[30] Ponto KA, Pitz S, Pfeiffer N, Hommel G, Weber MM, Kahaly GJ (2009). Quality of life and occupational disability in endocrine orbitopathy. Dtsch. Arztebl. Int..

