# Supplementary material for: Teprotumumab for the treatment of chronic thyroid eye disease
Source: Eye (Lond). 2021 Jul 9;36(8):1553–9. doi: 10.1038/s41433-021-01593-z (PMC9307784; doi:10.1038/s41433-021-01593-z)
Supplement: Supplementary file 1 — Table 2 [file 41433_2021_1593_MOESM1_ESM.docx]

|  | **Age** | **Gender** | **Ethnicity** | **Smoking History** | **Duration of TED Prior to first Infusion (Months)** | **Previous Treatments for TED** | **Thyroid Status at time of initial infusion** | **No. of Infusions** |
| --- | --- | --- | --- | --- | --- | --- | --- | --- |
| **Case 1** | 69 | Female | Caucasian | No | 36 | Decompression OS, 1 year prior to infusion | Euthyroid | 8 |
| **Case 2** | 69 | Female | Caucasian | No | 25 | none | Euthyroid | 8 |
| **Case 3** | 73 | Male | Caucasian | Former | 24 | IV corticosteroids | Euthyroid | 3 |
| **Case 4** | 29 | Female | Caucasian | No | 82 | IV corticosteroids | Euthyroid | 8 |
| **Case 5** | 33 | Female | Caucasian | No | 36 | Decompression OU 2 years prior to initiation of therapy | Euthyroid | 8 |
| **Case 6** | 68 | Female | Caucasian | No | 118 | IV corticosteroids | Euthyroid | 8 |
| **Case 7** | 35 | Male | Hispanic | No | 47 | none | Euthyroid | 8 |
| **Case 8** | 48 | Female | Caucasian | No | 67 | Decompression OU 3 years prior to initiation of therapy | Euthyroid | 5 |
| **Case 9** | 34 | Female | Asian | No | 38 | none | Euthyroid | 8 |
| **Case 10** | 38 | Female | Asian | No | 27 | none | Euthyroid | 7 |
| **Case 11** | 57 | Female | Caucasian | No | 36 | Decompression OS 2 years prior to infusion | Euthyroid | 7 |
| **Case 12** | 51 | Female | Caucasian | No | 144 | none | Euthyroid | 7 |
| **Case 13** | 35 | Female | Caucasian | No | 48 | none | Euthyroid | 5 |
| **Case 14** | 76 | Female | Caucasian | No | 184 | IV Steroids, strabismus surgery and bilateral orbital decompression | Euthyroid | 7 |
| **Case 15** | 67 | Female | Caucasian | No | 180 | Nil | Euthyroid | 5 |
| **Case 16** | 76 | Female | Caucasian | No | 84 | IV corticosteroids | Euthyroid | 8 |
| **Case 17** | 72 | Male | Black | No | 48 | Bilateral decompression, oral corticosteroids | Euthyroid | 8 |
| **Case 18** | 70 | Female | Caucasian | No | 204 | Orbital radiation, oral corticosteroids, bilateral decompression | Euthyroid | 8 |
| **Case 19** | 56 | Male | Caucasian | No | 156 | Orbital Radiation, oral corticosteroids | Euthyroid | 4 |
| **Case 20** | 55 | Female | Caucasian | No | 96 | Oral corticosteroids, orbital radiation | Euthyroid | 6 |
| **Case 21** | 57 | Male | Black | No | 120 | Oral corticosteroids, IV steroids, orbital radiation, bilateral decompression | Euthyroid | 8 |
| **Case 22** | 79 | Male | Black | No | 48 | Orbital decompression OD | Euthyroid | 8 |
| **Case 23** | 75 | Female | Caucasian | Former | 120 | Oral and IV corticosteroids, bilateral orbital decompression | Euthyroid | 8 |
| **Case 24** | 30 | Female | Hispanic | No | 24 | Oral corticosteroids, orbital radiation, decompression | Euthyroid | 8 |
| **Case 25** | 45 | Male | Asian | Former | 24 | IV corticosteroids, orbital radiation | Euthyroid | 8 |
| **Case 26** | 73 | Female | Hispanic | Former | 129 | IV and oral corticosteroids, orbital radiation | Euthyroid | 6 |
| **Case 27** | 51 | Female | Hispanic | Never | 26 | none | Euthyroid | 8 |
| **Case 28** | 53 | Female | Hispanic | Never | 54 | IV corticosteroids | Euthyroid | 5 |
| **Case 29** | 47 | Female | Asian | Never | 25 | none | Euthyroid | 4 |
| **Case 30** | 68 | Male | Black | Former | 108 | IV and oral corticosteroids, radiation, bilateral decompression, strabismus surgery | Euthyroid | 8 |
| **Case 31** | 71 | Female | Caucasian | Never | 156 | Oral corticosteroids and orbital radiation | Euthyroid | 4 |

**Table 1:** Demographic and clinical details
