# Supplementary material for: Teprotumumab for the treatment of chronic thyroid eye disease
Source: Eye (Lond). 2021 Jul 9;36(8):1553–9. doi: 10.1038/s41433-021-01593-z (PMC9307784; doi:10.1038/s41433-021-01593-z)
Supplement: Supplementary file 2 — Table 3 [file 41433_2021_1593_MOESM2_ESM.docx]

|  |  | **OD** | | | | **OS** | | | |
| --- | --- | --- | --- | --- | --- | --- | --- | --- | --- |
|  | **Case** | **Upgaze** | **Downgaze** | **Right** | **Left** | **Upgaze** | **Downgaze** | **Right** | **Left** |
| **Pre** | **1** | 0 | 0 | 0 | 0 | -4 | 0 | -0.5 | -2 |
| **Post** | **1** | 0 | 0 | 0 | 0 | -3 | 0 | 0 | -2 |
|  | 3 | -3 | 0 | -0.5 | -0.5 | -2 | 0 | -0.5 | -0.5 |
|  | 3 | -0.5 | 0 | -0.5 | -0.5 | -0.5 | 0 | -0.5 | -0.5 |
| **Pre** | **4** | 0 | 0 | 0 | 0 | 0 | -1 | 0 | 0 |
| **Post** | **4** | 0 | 0 | 0 | 0 | 0 | 0 | 0 | 0 |
|  | 6 | 0 | 0 | -2 | -1 | -2 | 0 | -1 | -2 |
|  | 6 | 0 | 0 | 0 | 0 | 0 | 0 | 0 | 0 |
| **Pre** | **8** | 0 | 0 | 0 | 0 | 0 | 0 | 0 | -1 |
| **Post** | **8** | 0 | 0 | 0 | 0 | 0 | 0 | 0 | -0.5 |
|  | 10 | 0 | 0 | 0 | 0 | -2 | 0 | 0 | -1 |
|  | 10 | 0 | 0 | 0 | 0 | -0.5 | 0 | 0 | 0 |
| **Pre** | **11** | -1 | 0 | -1 | 0 | -2 | 0 | 0 | -1 |
| **Post** | **11** | 0 | 0 | 0 | 0 | 0 | 0 | 0 | 0 |
| **Pre** | **15** | 0 | 0 | 0 | 0 | 0 | 0 | 0 | 0 |
| **Post** | **15** | 0 | 0 | 0 | 0 | 0 | 0 | 0 | 0 |
| **Pre** | **16** | 0 | 0 | 0 | 0 | 0 | 0 | 0 | 0 |
| **Post** | **16** | 0 | 0 | 0 | 0 | 0 | 0 | 0 | 0 |
| **Pre** | **17** | -3 | 0 | 0 | 0 | -1 | 0 | 0 | 0 |
| **Post** | **17** | -1 | 0 | 0 | 0 | 0 | 0 | 0 | 0 |
| **Pre** | **18** | 0 | 0 | -1 | 0 | -2 | 0 | -2 | -4 |
| **Post** | **18** | 0 | 0 | 0 | 0 | 0 | 0 | 0 | 0 |
| **Pre** | **19** | 0 | 0 | 0 | 0 | 0 | 0 | 0 | 0 |
| **Post** | **19** | 0 | 0 | 0 | 0 | 0 | 0 | 0 | 0 |
| **Pre** | **20** | -1 | 0 | 0 | -3 | 0 | 0 | 0 | 0 |
| **Post** | **20** | 0 | 0 | 0 | -3 | 0 | 0 | 0 | 0 |
| **Pre** | **21** | 0 | 0 | -1 | 0 | 0 | 0 | 0 | 0 |
| **Post** | **21** | 0 | 0 | -1 | 0 | 0 | 0 | -1 | 0 |
| **Pre** | **22** | -3 | -2 | 0 | -3 | -4 | 0 | -1 | 0 |
| **Post** | **22** | -3 | -2 | 0 | -3 | -4 | 0 | -1 | 0 |
| **Pre** | **23** | 0 | 0 | 0 | -2 | 0 | 0 | -1 | 0 |
| **Post** | **23** | -1 | 0 | 0 | -2 | 0 | 0 | -1 | 0 |
| **Pre** | **24** | 0 | 0 | 0 | 0 | -1 | 0 | 0 | 0 |
| **Post** | **24** | 0 | 0 | 0 | 0 | 0 | 0 | 0 | 0 |
| **Pre** | **25** | 0 | -4 | -2.5 | 0 | -4 | 0 | -1 | -4 |
| **Post** | **25** | 0 | -4 | -1 | 0 | -4 | 0 | 0 | -4 |
| **Pre** | **26** | 0 | 0 | -0.5 | 0 | -1 | 0 | 0 | -0.5 |
| **Post** | **26** | 0 | 0 | 0 | 0 | -0.5 | 0 | 0 | 0 |
| **Pre** | **27** | 0 | 0 | 0 | 0 | 0 | 0 | 0 | 0 |
| **Post** | **27** | 0 | 0 | 0 | 0 | 0 | 0 | 0 | 0 |
| **Pre** | **28** | -0.5 | 0 | 0 | 0 | -0.5 | 0 | 0 | 0 |
| **Post** | **28** | 0 | 0 | 0 | 0 | 0 | 0 | 0 | 0 |
| **Pre** | **29** | -2 | 0 | -1 | 0 | 0 | -1 | 0 | -1 |
| **Post** | **29** | 0 | 0 | 0 | 0 | 0 | 0 | 0 | -0.5 |
| **Pre** | **30** | 0 | 0 | -1 | -2 | 0 | 0 | 0 | 0 |
| **Post** | **30** | 0 | 0 | -1 | 0 | 0 | 0 | 0 | -1 |
| **Pre** | **31** | -1 | 0 | 0 | -1 | -1 | 0 | -1 | 0 |
| **Post** | **31** | 0 | 0 | 0 | -1 | 0 | 0 | -1 | 0 |

Table 3: Five-point strabismus scale assessing gaze of each eye in different directions, pre and post therapy (0 = no restriction, -1 = 25% restriction, 2 = 50% restriction, 3 = 75% restriction and 4 = no movement)
