# Supplementary material for: Teprotumumab for the treatment of chronic thyroid eye disease
Source: Eye (Lond). 2021 Jul 9;36(8):1553–9. doi: 10.1038/s41433-021-01593-z (PMC9307784; doi:10.1038/s41433-021-01593-z)
Supplement: Supplementary file 3 — Table 4 [file 41433_2021_1593_MOESM3_ESM.docx]

|  | **Before Treatment** | | | | | | **Post Treatment** | | | | | |
| --- | --- | --- | --- | --- | --- | --- | --- | --- | --- | --- | --- | --- |
| **Case**  **No.** | **Muscle Volume OD** | **Muscle Volume OS** | **Fat Volume OD** | **Fat Volume OS** | **Hertel OD** | **Hertel OS** | **Muscle Volume OD** | **Muscle Volume OS** | **Fat Volume OD** | **Fat Volume OS** | **Hertel OD** | **Hertel OS** |
| **1** | 2198 | 5214 | 8549 | 6846 | 18 | 19 | 2068 | 2928 | 8039 | 4744 | 16 | 14 |
| **4** | 3486 | 5487 | 11705 | 8643 | 20 | 25 | 1941 | 3584 | 6207 | 6849 | 17 | 20 |
| **6** | 3692 | 3714 | 10776 | 10145 | 24 | 24 | 3120 | 2997 | 8967 | 8778 | 20 | 20 |
| **7** | 4897 | 4873 | 19285 | 24039 | 21 | 24 | 2425 | 2783 | 19695 | 19763 | 19 | 21 |
| **9** | 3571 | 3712 | 10231 | 10362 | 23 | 24 | 2701 | 2957 | 8754 | 8432 | 18 | 19 |
| **14** | 4330 | 4045 | 8315 | 9316 | 28 | 27 | 3513 | 3251 | 7445 | 8527 | 26 | 25 |
| **17** | 7389 | 8795 | 16592 | 15845 | 29 | 28 | 4724 | 7046 | 15013 | 15829 | 25 | 26 |
| **18** | 4983 | 6392 | 17180 | 14263 | 28 | 25 | 6064 | 4071 | 13998 | 11587 | 20 | 17 |
| **19** | 4872 | 5196 | 18466 | 18561 | 25 | 25 | 4827 | 4962 | 18328 | 18060 | 24 | 24 |
| **20** | 2335 | 2142 | 14808 | 15087 | 22 | 20 | 2330 | 1847 | 14519 | 13173 | 19 | 17 |
| **21** | 8523 | 8706 | 19377 | 24471 | 30 | 30 | 7914 | 6783 | 16697 | 23147 | 22 | 25 |
| **22** | 11904 | 9834 | 13209 | 15792 | 24 | 30 | 8433 | 7106 | 10004 | 11164 | 15 | 25 |
| **23** | 12102 | 10973 | 18726 | 18241 | 35 | 35 | 7714 | 7491 | 17963 | 17725 | 33 | 31 |
| **30** | 13018 | 14736 | 14637 | 12988 | 32 | 32.5 | 2927 | 3144 | 12418 | 12964 | 26 | 28 |
| **31** | 3156 | 2998 | 14990 | 13767 | 18 | 19 | 8433 | 7106 | 10004 | 11164 | 18 | 15 |

Table 4: Muscle and Fat volume pre and post teprotumumab therapy
